# A new multimedia cryptosystem using chaos, quaternion theory and modular arithmetic

**DOI:** 10.1007/s11042-023-14475-1

**Published:** 2023-03-09

**Authors:** Mendez Luis, Ladino Daniel, Amaya Isabel, Alvarado Deicy

**Affiliations:** Complexity Research Group (COMPLEXUD), Engineering Faculty, Universidad Distrital Francisco Jose de Caldas, Bogota, 110231 Colombia

**Keywords:** Quaternions, Chaos, Cryptosystem, Multimedia, Modular arithmetic, Residual matrices

## Abstract

Based on the combination of quaternion numbers, residual matrices, and chaotic attractors, a new cryptosystem is proposed for multimedia processing files such as images and audio. The key employed in this encryption schema consists of an image with a wide and sensitive range, obtained from the Julia Quaternion set rendered using a computational tool. Due to the use of quaternion matrices mixing the information between RGB layers and audio samples was possible, whereas using XOR operation and residual matrices modulus 257, added high sensitivity to small perturbations during encryption, key preparation and decryption processes, to such an extent that a minimal change in the image or in the audio leads to a totally different encryption result. The use of dynamic programming also reduced the processing time for matrix operations on the $\mathbb {Z}_{257}$ ring. To corroborate security of the algorithm, different tests were performed, including the National Institute of Standards and Technology test obtaining different indicators that were compared with other scientific references of similar works, finding behavioral patterns in accordance with those referenced works.

## Introduction

During recent times consumption of multimedia on Internet has increased drastically, COVID-19 pandemic has also contributed in this sense, reason why the scientific community continues developing mathematical and engineering strategies that lead to cryptographic solutions each time more secure and efficient. In the current work, quaternions, chaotic attractors and modular arithmetic are employed in order to design a cryptographic scheme focused on multimedia files (audio and images).

### Multimedia and traditional schemes

The increase in information volume exchange through public channels has raised the need to protect data confidentiality. The ever-increasing flux of multimedia files on the Internet has been reported by FileCatalyst (a firm responsible for providing software services to speed up and optimize the transmission of multimedia files), showing the range of sizes for different types of multimedia files in 2020. This data is consolidated in Table 1. The table illustrates an exponential increase in file size regarding new technologies, and this trend is expected to continue with the arrival of new developments such as 8K and augmented reality.

**Table 1:** Sizes of different multimedia formats in MB

File Type	Size (MB)
E-Book	1–5
MP3	3.5
CD-ROM	750
DVD(Movie)	4.096
Full-HD Movie	8.192–15.360
Blue-Ray Movie	20.480–25.534
4k Movie	102.400–200.000
8k Movie	553.000+

Source: Own elaboration

The Canadian company Sandvine, specialized in providing solutions for network policies and control of landline and mobile communications services, with emphasis on cyber security, in its annual report, shows that multimedia consumption all over the world had reached almost 45% in 2018 and 60% in 2020, so therefore it is necessary to strengthen encryption schemes for multimedia files by providing academic solutions that are applicable to the industry [10].

Growing concern for the protection of information led to traditional encryption schemes such as Advanced Encryption Standard (AES) and Data Encryption Standard (DES) emerged, however, due to limitations such as the handling of high-volume information, low levels of entropy and the high correlation of data, these traditional algorithms are less suitable for multimedia encryption [14, 27]. The performance of AES leads to redundancy when applied to large files, which has prompted the proposal of new specialized approaches for multimedia files such as those based on elliptical curves [6, 40], chaotic attractors, quantum computing, DNA code [38, 51, 53], commutative and non-commutative rings, cellular automatons [5] and Visual Cryptography-Based Watermarking [36].

### Quaternions and cryptography

Quaternion numbers have a non-commutative ring structure, which makes them useful in cryptography and image processing, since RGB channels are handled jointly by each quaternion component and rotations require less computational processing compared to their vectorial counterpart.

These numbers have been use in the last decade as a language in a variety of cryptography applications such as security schemes for multimedia files, key generation, public key algorithms, digital signatures, and hashing. Some related works are described below.

In [9, 17], an image, audio and text encryption model is proposed using Quaternion Fourier Transform (DQFT). Regarding key generation proposals, the work in [4] uses Julia-Quaternion set to generate keys in real time. Other studies, such as [15, 41], and [20], use Quaternion polynomials, modular operations and powers of Quaternion numbers to produce different public key algorithms. In [48] and [30], hashing and security schemes over a Wi-fi network are designed taking advantage of Quaternion rotations. Other works that utilize quaternions for handling pixel rotations in images can be found in [8, 12, 14, 49, 52].

A meaningful work in the medical sector was proposed in [13], the authors intended to achieve a balance between security level and performance of a cryptographic algorithm suitable for handling large volumes of medical information. In order to achieve this, they relied on quaternion theory and modular arithmetics, proposing a model focused on the secure handling of digital images and communication for DICOM medicine, in this case, taking advantage of quaternion rotation mechanism (which requires minimal computational cost) defining a Feistel-type encryption process on which for each iteration a different quaternion encryption key was used.

However, authors subsequently identified a weaknesses in their original proposal, that led them to design an encryption model with similar characteristics at [12] trying to address the vulnerabilities found in their previous work. Essentially, they define a new mechanism for key generation given the Julia quaternión set, preserving the rotation scheme and the Feistel-type encryption process used in their original algorithm.

### Chaos and cryptography

Moreover, chaos properties are very useful in cryptographic systems because its applications comprehend from key generation and system synchronization schemas to pseudo-random number generation algorithms.

There are also works focused on application of logical operations such as XOR to provide new security layers based on pseudo-random number generators (PRNG); such is the case of [37], in which the XOR operation is used between the adjacent pixels of a gray-scale image, leading to an increase in security of the scheme. Likewise, in [34], three pseudo-random number generation algorithms, using XOR and AND logic gates, cleared successfully all the security tests associated to the NIST sts-2.1.1 standard (a standard by the National Institute of Standards and Technology).

In [35], a Lorentz attractor is used to encrypt images in gray scale, on the other hand similar works like [14, 44] use the same attractor adding one more dimension in order to increase the complexity and encrypt color pictures. Authors such as Min, Zhang, and Zhang [28] also proposed a pseudo-random number generator (PRNG) that complied with the metrics defined by the Federal Information Processing Standard 140-2 (FIPS) based on two chaotic attractors of three and six dimensions.

Likewise in [50], they use Rossler and Lorentz attractors in order to establish an image permutation and diffusion scheme by applying a zigzag and spiral scanning technique to pixels from original image and encrypting them using ElGamal algorithm. As a result of this approach, they reported security and performance indicators enclosed to similar encryption approaches.

In this direction, proposals presented by [18] and [27] on which they take advantage of two dimensional fractal properties like Mandelbrot’s and Julia’s set, are clear evidence that it is possible to propose new chaos based cryptosystems with security measures closer to those of traditional schemes.

For example, works proposed in [2, 21, 23–25, 31, 33, 39, 46, 47, 55, 56] focus on the algorithm design for image encryption using different chaotic attractors of one or more dimensions, security and performance results demonstrate them to be security effective in different fields.

Moreover, although in [26, 29, 32] they develop chaos based algorithms for image encryption, they deserve to be noticed because of the use of DNA encryption, on which they achieve convincing results in cryptography.

### Modular arithmetic and cryptography

Another interesting concept that has been applied in cryptography and science is the use of residual classes, leading among other things to formalization of residual matrix theory, such concept originated with the proposal of Rabin’s asymmetric cryptosystem, and has evolved thanks to the introduction of the latest generation of computers. At [16] they suggest the use of residual classes to propose a modification of Rabin’s asymmetric model applied to text message encryption, achieving an increase in blocks size used by the original model. Moreover, they consolidate three algorithms which through hardware implementation shows that runtime is reduced by approximately half in comparison with the original Rabin’s method, thanks to a more efficient way to calculate modular powers and roots.

### About this approach

Based on related works as well as on approaches found in [4, 14–17], this paper proposes the use of Quaternion theory with residual matrices in order to design a new cryptosystem intended for multimedia files. The cryptosystem uses a chaotic dynamic system to obtain an image that serves as a private key. Parameters for private key generation are forwarded through a public key algorithm. The cryptosystem involves several layers of protection including modular arithmetic, XOR operation and quaternion product.

An advantage of this paper lies in the use of a quaternion matrix to perform simultaneous operations between RGB layers and audio samples which are handled through the quaternion real component, decreasing the processing of diffusion and permutation operations as they are performed using parallel computing.

When images are generated from chaotic systems with 3 or more dimensions, a huge number of possibilities are added to guarantee the uniqueness of each image, such as its initial conditions (sensitivity), cutting planes, iterations (periodicity), texture material of the chaotic system, colors, patterns, objects in space, their physical properties (shape, reflection rates, patterns, colors) and how they interact, all of these guarantee a large and secure key space.

If an equally unique sequence of pseudo-random numbers can be created from each unique rendered image that is sensitive to even the smallest bit change, then it will serve as a key in a cryptosystem. In this proposal, the key image is obtained from Julia’s quaternion set whereby its dynamic nature constitutes an advantage in key’s security.

On the other hand, modular inverse 257 is employed because adding the unity to all RGB values in each component results in a range between [1,256], thus guaranteeing the uniqueness of modular inverse, this fact is exploited in order to increase pixel’s sensitivity and ensure that the slightest change in key image generates a different bit-stream. Additionally, the algorithm is designed in such way that every key’s pixel is relevant to the whole encryption process.

In order to safely manipulate multimedia information of this proposal, the encryption mechanism handles quaternion matrix multiplication with integer entries, and also relies in modulus 257 residual classes calculations through parallel processing and dynamic programming, simplifying the computational cost which is an advantage related to computational complexity.

The necessary concepts are explained in Section 2, followed by the proposed model in Section 3, after this, implementation is shown in Section 4, followed by the different test and complexity analysis in Section 5, and finally conclusions are drawn in Section 6.

## Preliminaries

The cryptosystem proposed in this paper involves two main components, encryption algorithm and key generation. The theoretical basis for the proposed algorithm is described below.

### Quaternion numbers

The Quaternion number set *H* is defined by three imaginary units *i*,*j*,*k*; a Quaternion *q* has a form as shown in (1):

1 $$ \begin{array}{@{}rcl@{}} q=a+ib+jc+kd=(a,v), \quad  a,b,c,d \in \mathbb{R} \end{array} $$

where *v* is vector *v* = (*b*,*c*,*d*); *a* and *v* are named real and vectorial part of Quaternion respectively. If the real part of Quaternion is zero, the resulting number is known as a pure Quaternion.

On Quaternion set *H*, addition and multiplication operations are defined and fulfill the structure of a non-commutative division ring (the cryptosystem proposal takes advantage of this) [19].

### Residual matrices

The notion of congruence was given by Gauss and dates back to 1801. Specifically, given *x*,*y*,*m* positive integers, *x* is congruent with *y* module *m* (represented by (2)) if *m* divides the number *x* − *y*.

2 $$  x \equiv y\mod m $$

This notion satisfies the properties of an equivalence relation, which eases the grouping of integers in disjunct families given the fact that two positive integers are congruent module *m* if and only if they are in the same family, they are known as residual classes module *m*, and they are designated by $\mathbb {Z}_{m}$. It should be noted that there are exactly *m* residual classes module *m*, and that $\mathbb {Z}_{m}$ has a commutative ring structure given the sum and product defined for them [18].

Residual classes have also brought up the need to solve congruential equations from the simplest ones like those of linear and quadratic type, to simultaneous congruence systems. This is where the Chinese Theorem of the remainder proposed by mathematician Sun Tzu comes into place as a powerful tool to find solutions for linear congruence systems which is also applied for solving polynomial congruencies for composite modules.

Theory of residual classes has been widely applied into cryptography mainly to reduce the number of operations through congruencies when dealing with excessively long values, as a consequence it results into a decrease of computational speed, making room for cryptographic model proposals based on them and the notion of residual and inverse matrices.

### Chaotic dynamical systems

The chaotic dynamical system definition adopted in this document was proposed in 1948 by Robert Devaney [11]. Chaos is an aperiodic behavior sensitive to initial conditions found in systems as they evolve in time. This definition corresponds to the existence of a *strange attractor*, as explained in dynamical systems literature. The chaotic behavior of such systems is an advantage in order to propose key generation schemes using Quaternions. Such proposal is possible due to the similarity between chaotic dynamical systems properties and permutation/diffusion features for cryptographic systems.

**Fig. 1:**
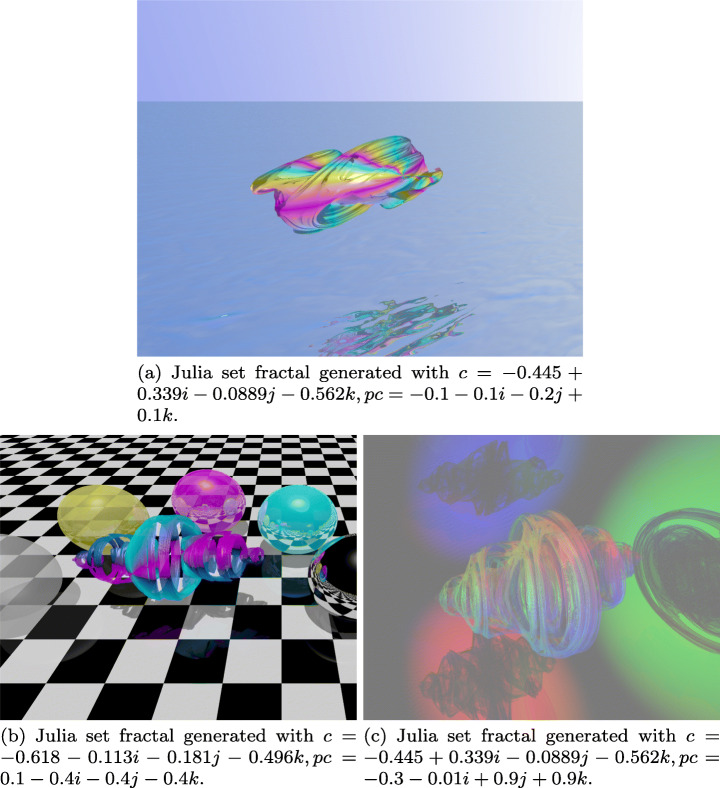
Julia fractals generated. Source: Own elaboration with PovRay

An example of a chaotic system can be seen by iterating the quadratic function *f*(*z*) = *z*^2^ + *c* over the Quaternion set, which results into the Julia Quaternion set (in honor of mathematician Gaston Julia), where *c* is an arbitrary parameter. Figure 1 shows a few of many fractals that can be generated from this chaotic attractor, which can also be enriched with effects typically found in a three-dimensional space such as illumination, shadows, reflections and transparencies.

## Proposed model

**Fig. 2:**
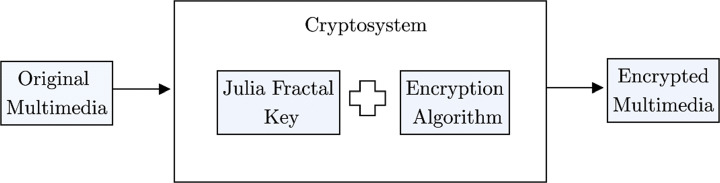
Proposed Model. Source: Own elaboration

This paper proposes an encryption system for the processing of multimedia files based on Quaternion numbers set, Julia Quaternion chaotic attractor, and residual matrices. Figure 2 gives an overview of the cryptosystem. In order to generate the cryptosystem’s key as an image, a software known as PovRay was used, in which several parameters are transmitted through an insecure channel using a public key algorithm. Additionally, a cryptographically secure pseudo-random number generator (CSPRNG) should be also implemented in order to increase the security level.

### Key generation

The key generated was obtained from Julia Quaternion set on which a 4-dimensional fractal is yield and intercepted by a cutting plane in order to retrieve a $\mathbb {R}^{3}$ fractal that results in the key for encryption/decryption. Thanks to PovRay, an open source software, the key was easily generated because it provides users with a native Julia Quaternion set implementation. As another interesting feature of the software it also allows parameters adjustments such a *c* in *f*(*z*) function, number of iterations, the cutting plane, the camera location, as well as visual parameters like lighting, rendering texture, image background, among others.

**Fig. 3:**
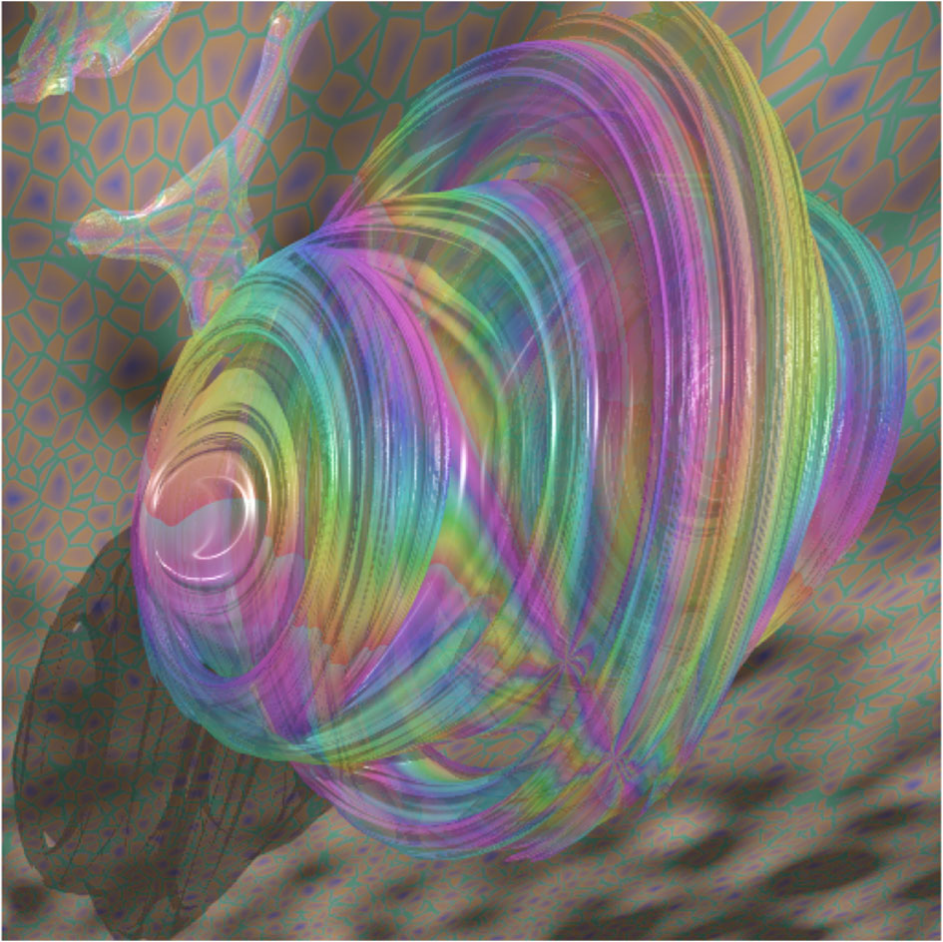
Key example obtained with *c* = − 0.310 − 0.0410*i*− 0.325*j*+ 0.560*k*,*pc* = 0.1 + 1*i*+ 1*j*+ 1*k*,*n* = 11,*RGB*_1_ = (0.2,0.6,0.4),*RGB*_2_ = (0.5,0.5,0.6),*RGB*_3_ = (0.8,0.47,0.2),*RGB*_4_ = (0.15,0.20,0.8),512×512,*Fr* = 8. Source: Own elaboration with PovRay

In this paper, function *f*(*z*) = *z*^2^ + *c* was used with *c* and *z* both being Quaternions. The cutting plane is denoted as *pc* and the number of iterations corresponds to *n*. Four RGB vectors were used for color depth. The radial frequency is denoted as *F*_*r*_ and the size of the multimedia object to be encrypted is specified in all cases. An example of a key obtained from PovRay is shown in Fig. 3.

### Key distribution

In this paper, the cryptosystem implemented 9 independent sub-keys in order to send all the necessary key parameters. Each parameter uses a different key denoted by *k*_*i*_, where *i* refers to the required key generation parameters to be sent.

The parameters in question are as follows: Julia fractal generator constant *c*; cutting plane *pc*; random generated Quaternions that are associated to the RGB color percentage for background texture *RGB*_1_, *RGB*_2_, *RGB*_3_ and *RGB*_4_; Quaternion *p*, which is the combination of the radial frequency *F*_*r*_, number of iterations, *n* and two *M*, *N* values corresponding to the image height and width; Quaternion *t* which is composed from the audio sample, frequency *as* for framerates and snake algorithm displacement *snk*; and finally a Quaternion *s*, which corresponds to the CSPRNG seed. The key is then obtained from (3) to (11).

3 $$  k_{c}=c $$

4 $$  k_{pc}=pc $$

5 $$  k_{RGB_{1}}=RGB_{1} $$

6 $$  k_{RGB_{2}}=RGB_{2} $$

7 $$  k_{RGB_{3}}=RGB_{3} $$

8 $$  k_{RGB_{4}}=RGB_{4} $$

9 $$  k_{p}=p $$

10 $$  k_{s}=s $$

11 $$  k_{t}=t $$

In order to avoid the discrete logarithm problem, various public key proposals have been introduced using Quaternions for key parameters distribution [15, 20, 41].

### Encryption model

The encryption process is four-fold:

- Key preparation.
- Quaternion matrix generation.
- Residuals matrix multiplication
- Quaternion Product Calculation
- Inverse residual matrix multiplication

**Fig. 4:**
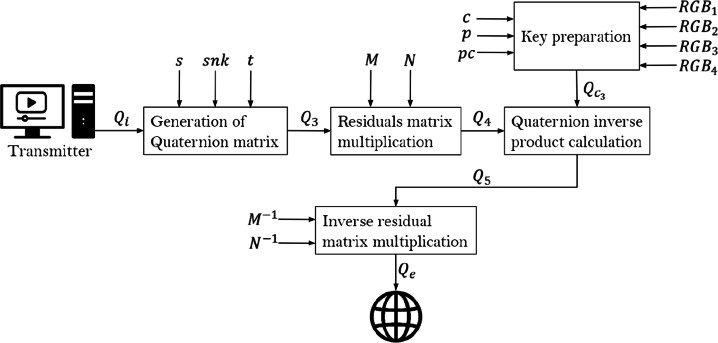
Encryption scheme. Source: Own elaboration

Figure 4 illustrates the encryption process.

#### Key preparation

With the aim of ensuring the best entropy and sensitivity to small changes, the following steps were included in order to prepare the key for encryption process:

1. Julia quaternion fractal key image is rendered from parameters identified in (3) to (9), which must be sent to the receiver to retrieve the original information.
2. Decompose the key image into its RGB components $Q_{c_{0}}=R_{c}i+G_{c}j+B_{c}k= (r_{c_{0ij}} i + g_{c_{0ij}} j + b_{c_{0ij}} k)$, where $r_{c_{0ij}}$, $g_{c_{0ij}}$, $b_{c_{0ij}}$ are the pixels values *ij* with *i* = 1,...,*n* *j* = 1,...,*m* at layers *R*, *G*, *B*, respectively.
3. Add matrix $Q_{c_{0}}$ to a pure quaternion matrix *A* having all of its entries equal to one in order to suppress possible zero values that may exist and interfere with the modular operation of next step thus obtaining $Q_{c_{1}}=Q_{c_{0}}+A= (r_{c_{ij}} i + g_{c_{ij}} j + b_{c_{ij}} k)$.
4. Define matrix *D* = (*d*_*ij*_) which is obtained by applying operation mod 257 to the product of each component in *Q*_*c*1_ at *ij* position, in other words $d_{ij}=(r_{c_{ij}} g_{c_{ij}} b_{c_{ij}}) \mod 257$.
5. Calculate the (mod 257) modular inverses for each RGB layer entries present in matrix *Q*_*c*1_ noted by $r_{c_{ij}}^{-1}$, $g_{c_{ij}}^{-1}$, $b_{c_{ij}}^{-1}$, to obtain matrix $Q_{c2}=(x_{ij} i +y_{ij} j+ z_{ij} k)= (q_{c_{ij}})$ synthesized in (12).
6. In case that (||*Q*_*c*2_||) mod 257 = 0 as a consequence of adding the value *d*_*ij*_, zeros are removed from matrix *Q*_*c*2_, applying function at (13) element by element obtaining $Q_{c3}=(p_{c_{ij}})$.

12 $$  \left\{\begin{array}{c} x_{ij}=(r_{c_{ij}}^{-1} g_{c_{ij}}+d_{ij}) \mod 257 \\ y_{ij}=(g_{c_{ij}}^{-1} b_{c_{ij}}+d_{ij}) \mod 257 \\ z_{ij}=(b_{c_{ij}}^{-1} r_{c_{ij}}+d_{ij}) \mod 257 \end{array}\right. $$

13 $$  p_{c_{ij}} =\left\{\begin{array}{cc} (x_{ij}^{2} i+ y_{ij}^{2} j + z_{ij}^{2} k) \mod 257 , & \left | q_{c_{ij}} \right | = 0 \\ q_{c_{ij}} & , \left | q_{c_{ij}} \right | \neq 0 \end{array}\right. $$

In summary, the proposed algorithm key is given by quaternion matrix *Q*_*c*3_.

#### Quaternion matrix generation

**Fig. 5:**
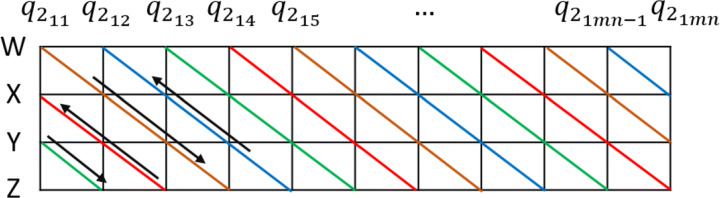
Snake algorithm, *snk* = 0. Source: Own elaboration

Depending on multimedia type, one of the following processes is carried out:

- **For images**: RGB image is decomposed into layers given *Q*_*i*_ = *a* + *Ri* + *Gj* + *Bk* = (*r*_*ij*_*i* + *g*_*ij*_*j* + *b*_*ij*_*k*), in this case *a* = 0, subsequently the original image matrix is masked by a quaterion matrix with random integer entries *Q*_*m*_ obtained from a CSPRNG instantiated by seed *s* specified at (10), this results in matrix $Q_{1}=(Q_{i}+Q_{m})\mod 256=(q_{1_{ij}})$, which is then arranged in quaternion vectors $Q_{2}=(q_{2_{1j}})$ in $\mathbb {R}^{nm}$ for *j* = 1,2,...,*mn*. In order to increase the cryptosystem’s small changes sensitivity, an algorithm is applied on *Q*_2_ obtaining matrix *Q*_3_ = *snake*(*Q*_2_), which consists in the XOR operation application between vector’s adjacent quaternions, starting from *snk* vector position according to (11), but moving between the quaternion’s cartesian coordinates as shown in Fig. 5, a point to note is that arrows point out the trajectory orientation to be followed.
  In particular, along the brown trajectory, the sequence of operations performed is shown at (14).
  14 $$  \begin{array}{ccc} x(q_{2_{12}})=w(q_{2_{11}})\bigoplus x(q_{2_{12}}) & \quad y(q_{2_{13}})=x(q_{2_{12}})\bigoplus y(q_{2_{13}}) &\\ z(q_{2_{14}})=y(q_{2_{13}})\bigoplus z(q_{2_{14}}) & \quad w(q_{2_{15}})=z(q_{2_{14}})\bigoplus w(q_{2_{15}}) \end{array} $$
  Hence, result from previous XOR operation is then stored in the major index for further use at next iteration. On (15) the red trajectory sequence is presented at Fig. 5 which traverses from right to left, for this case, XOR operation result is stored in the minor index determined by trajectory direction.
  15 $$  \begin{array}{ccc} y(q_{2_{1mn-1}})=z(q_{2_{1mn}})\bigoplus y(q_{2_{1mn-1}}) &  x(q_{2_{1mn-2}})=y(q_{2_{1mn-1}})\bigoplus x(q_{2_{1mn-2}}) &\\ w(q_{2_{1mn-3}})=x(q_{2_{1mn-2}})\bigoplus w(q_{2_{1mn-3}}) &  z(q_{2_{1mn-4}})=w(q_{2_{1mn-3}})\bigoplus z(q_{2_{1mn-4}}) \end{array} $$
  It is possible to generalize the XOR operation carried out along the four trajectories according to expressions shown at (16).
  16 $$  \begin{array}{ccc} \text{Left to right}\quad & \text{Right to left} \\ x=w\bigoplus x & x=y\bigoplus x\\ y=x\bigoplus y & y=z\bigoplus y \\ z=y\bigoplus z & z=w\bigoplus z\\ w=z\bigoplus w & w=x\bigoplus w \end{array} $$
- **For audio**: Given *as* value present at (11), the 32-bit samples are decomposed (it is also possible to adjust the audio for 64-bit samples) into 4 8-bits inputs and then stored in the quaternion real part *a* of *Q*_*i*_ = *a* + *Ri* + *Gj* + *Bk* = (*a*_*ij*_ + *r*_*ij*_*i* + *g*_*ij*_*j* + *b*_*ij*_*k*). If there is only an audio sample to be processed, an arbitrary white noise image is used in order to implement the same mask and snake process carried out for images.
- **For video**: A combination of the previous cases is applied. However, to ensure synchronization, audio component is divided into small samples according to the number of frames per second.

Up to this step multimedia information has been hidden in quaternion matrix *Q*_3_.

#### Residual matrix multiplication

Using matrix *Q*_3_ and two invertible residual square matrices *M*, *N* with *m* and *n* sizes respectively in $\mathbb {Z}_{257}$, the matrix multiplication described at (17) is applied.

17 $$  Q_{4}=(((Q_{3} M)^{T}N)^{T})\mod 257=(N^{T}Q_{3} M)\mod 257=(q_{4ij}) $$

The main purpose for calculations to happen in $\mathbb {Z}_{257}$ space is to reduce computational time and guarantee uniqueness of the inverse residual matrix, which is achieved by determinant *d* mod 257 being different than zero.

#### Quaternion product calculation

A modular operation is applied to the quaternion product between key $p_{c_{ij}}$ given by (13), its modular inverse $p_{c_{ij}}^{-1}$ and quaternion *q*_4*ij*_ present at (18).

18 $$  Q_{5}=(q_{5ij})=(p_{c_{ij}}^{-1}q_{4ij}p_{c_{ij}})\mod 257 $$

Taking advantage of quaternion numbers algebra, we generate matrix *Q*_5_ which is used at next item in order to add a security layer.

#### Inverse residual matrix multiplication

To end up the encryption process, residual matrices of item Section 3.3.3 are re-used but this time applying their residual inverses along with Matrix *Q*_5_, thus obtaining *Q*_*e*_ given by (19), which at the end is an array containing all original encrypted information and that is transmitted over the network in a secure way.

19 $$  Q_{e}=(N^{-1}(M^{-1}{Q^{T}_{5}})) \mod 257=(N^{-1}Q_{5}(M^{-1})^{T}) \mod 257 $$

### Decryption model

**Fig. 6:**
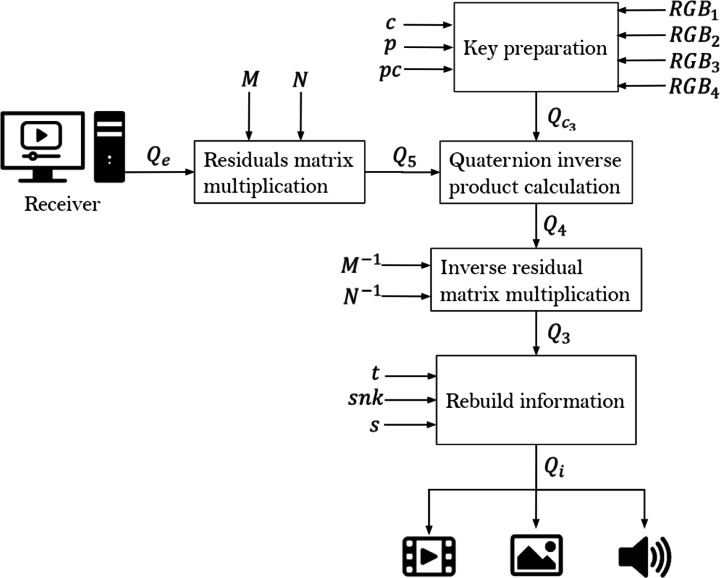
Decryption scheme. Source: Own elaboration

Based on matrix *Q*_*e*_, the decryption mechanism is similar to its encryption counterpart in reversed order, until matrix *Q*_*i*_ is obtained, which is shown in Fig. 6.

## Implementation

**Fig. 7:**
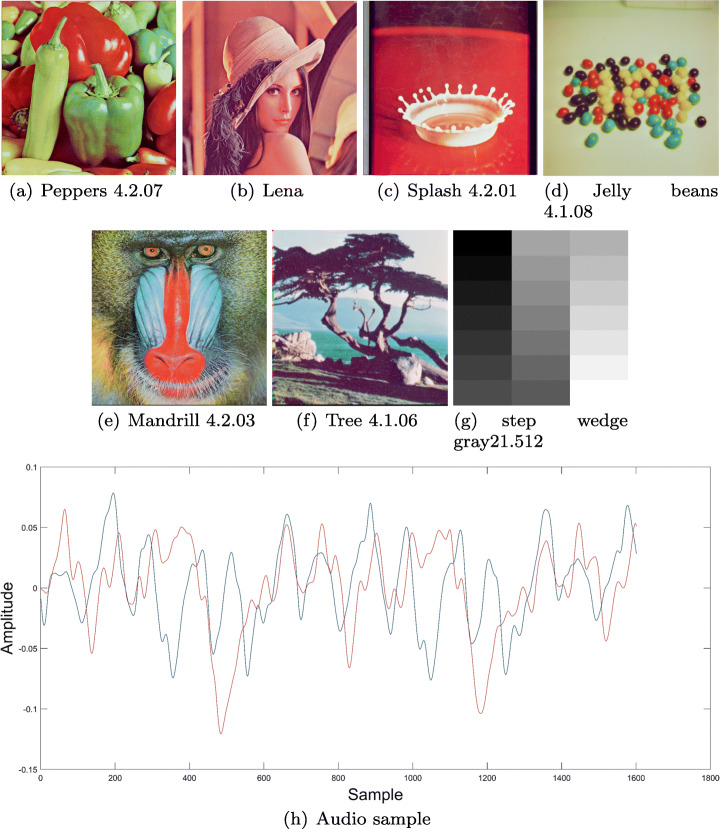
Encryption algorithm items. Source: USC-SIPI and Ludwig van Beethoven

Several tests were performed using the Lena picture and six different images from the USC-SIPI Image Database, as for the audio sample, Beethoven’s Moonlight Sonata musical composition from Ludwig van Beethoven was taken into consideration, finally the key presented in Fig. 3 was chosen for encryption/decryption. Images (a), (b), (c),(d), (e), (f) and (g) along with audio sample (h) are shown in Fig. 7. The implementation was performed using MATLAB Total Academic Headcount (TAH) license, and a computer whose specifications were: AMD Ryzen 1600x 3.6Ghz-3.9Ghz, Nvidia 2060 and 24 GB RAM at 3000 mHz.

**Fig. 8:**
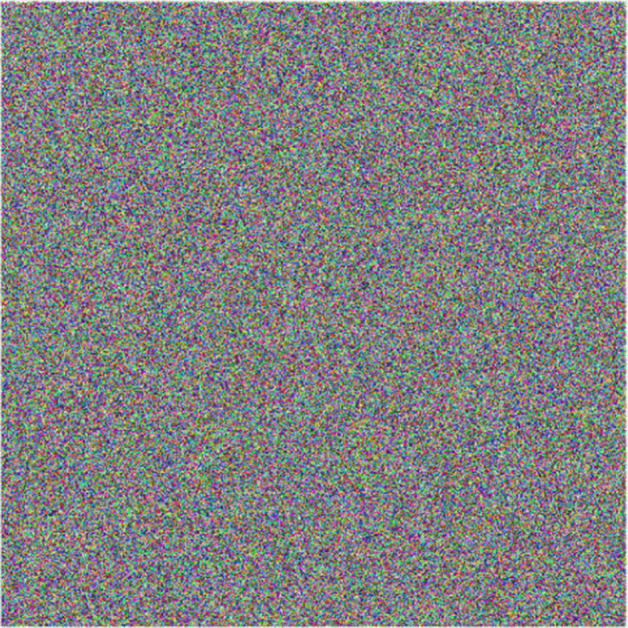
Key from Fig. 3 after key preparation process. Source: Own elaboration using MATLAB

**Fig. 9:**
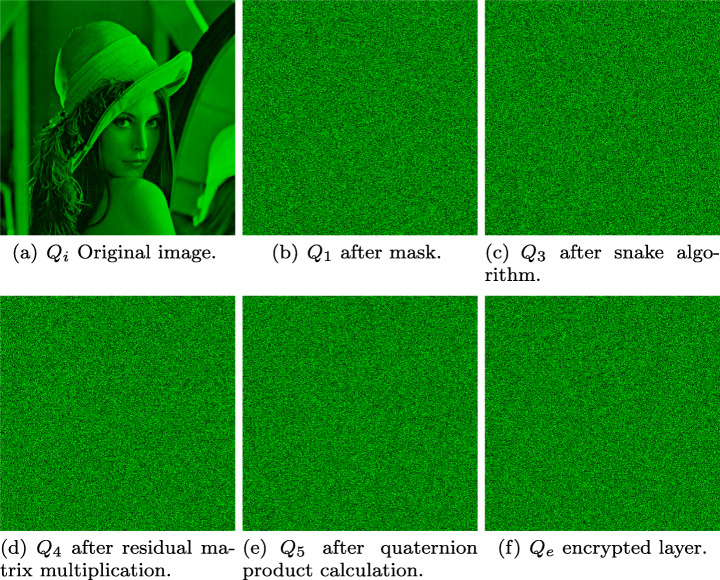
Green layer transition during encryption process. Source: Own elaboration using MATLAB

Following the proposed scheme, at first the key image preparation is performed, thus obtaining image at Fig. 8, afterwards, the encryption algorithm steps are executed sequentially on all layers. In order to show a particular application case, the Lena’s (Fig. 7(b)) green layer transformation over the encryption process is presented at Fig. 9, showing that under this approach the original information is hidden.

**Fig. 10:**
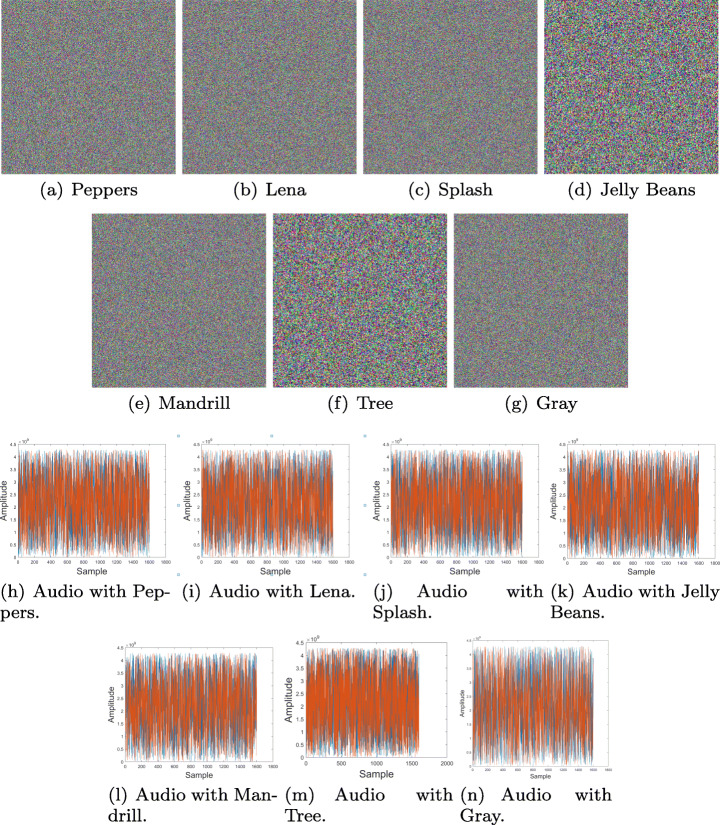
Encrypted multimedia. Source: Own elaboration using MATLAB

The final encryption results for the 7 sample images and audio sample correspond to the *Q*_*e*_ matrices and can be observed in Fig. 10. A point to note is that the encrypted audio sample varies according to the image employed in the process.

**Fig. 11:**
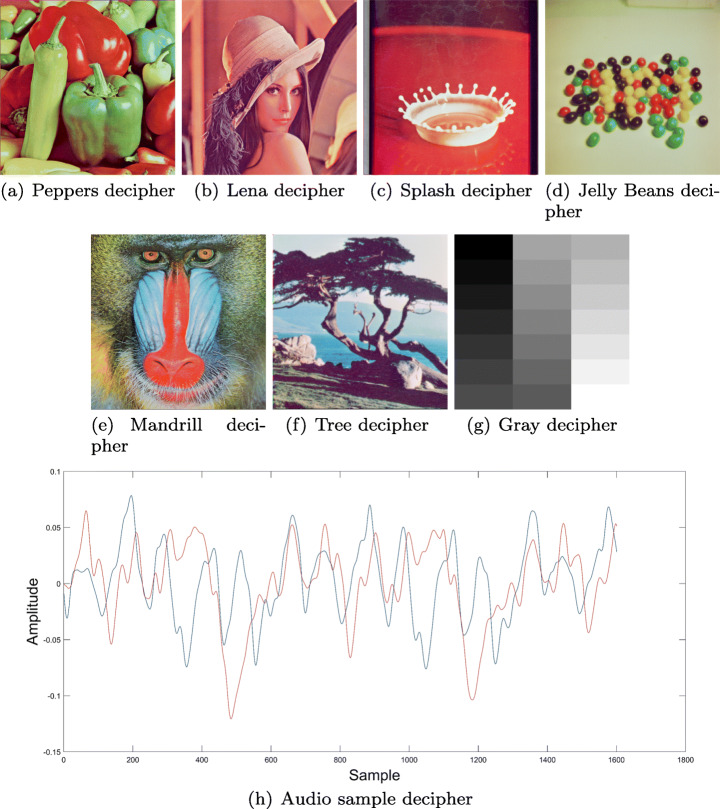
Algorithm items after decryption process. Source: Own elaboration using MATLAB

In order to recover the original information, elements from Fig. 10 are taken along with the prepared key from Fig. 8 and steps specified on Fig. 6 are executed, thus obtaining the original information shown at Fig. 11.

## Security analysis

The analysis applied to the proposed cryptosystem satisfies the National Institute of Standards and Technology (NIST) [7] specifications, which also considers histograms, correlation coefficients, differential analysis, key sensitivity and entropy values. The tests were performed using the images and audio sample presented in Fig. 7 and key shown at Fig. 8.

### NIST statistical test

This test consists of 15 different statistical measures that evaluate the chaotic bit-stream sequence on a statistical level, P-values establish whether a sequence pass the tests successfully or not, for this, such value must be greater than 0.01 at each one of the tests applied on *m* blocks out of *n* bits [7].

NIST tests on this work were performed with the “sts-2_1_2” Suite, by using as input file four-component data blocks corresponding to each quaternion matrix entry specified at (19), the library assessed 80 blocks out of 10^5^ bits, results presented at Table 2 are enough to state that the cryptosystem is statistically secure [14].

**Table 2:** NIST tests

	Pepper’s sample	Lena’s sample	Splash’s sample
Frequency	0.9292	0.7955	0.6371
Frequency Test within a Block	0.1223	0.0798	0.7399
Cumulative Sums	0.2133	0.6816	0.4372
Runs	0.3505	0.3741	0.9114
Test of the Longest Run of Ones in a Block	0.0510	0.0028	0.2757
Binary Matrix Rank Test	0.3987	0.1329	0.4372
Discrete Fourier Transform (Spectral)	0.6223	0.1441	0.2757
Non-overlapping Template Matching	0.7681	0.5341	0.5341
Overlapping Template Matching	0.0871	0.6222	0.5341
Maurer’s “Universal Statistical” Test	0.3925	0.9114	0.8343
Approximate Entropy	0.1692	0.6519	0.4372
Random Excursions	0.2757	0.6371	0.0251
Ramdom Excursions Variant	0.1626	0.0031	0.1626
Linear Complexity	0.2299	0.1223	0.5341
Serial	0.4777	0.5341	0.2133

### Histogram analysis and *χ*^2^ test

**Fig. 12:**
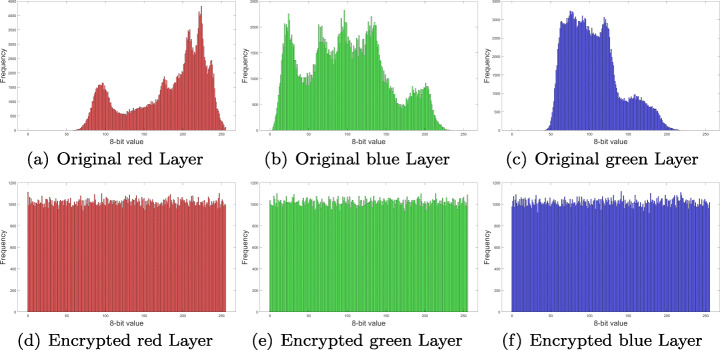
RGB layers histograms for Lena image from Fig. 7(b). Source: Own elaboration using MATLAB

**Fig. 13:**
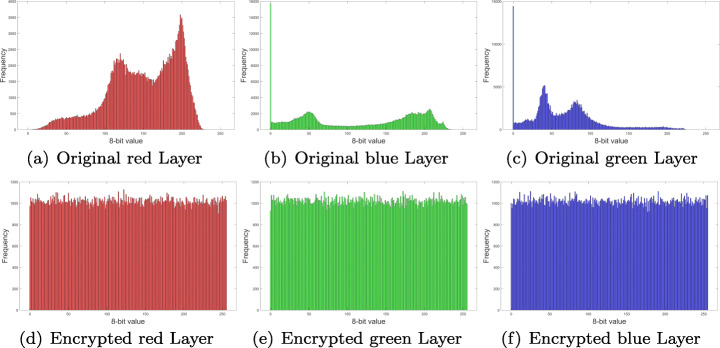
RGB layers histograms for Peppers image from Fig. 7(a). Source: Own elaboration using MATLAB

**Fig. 14:**
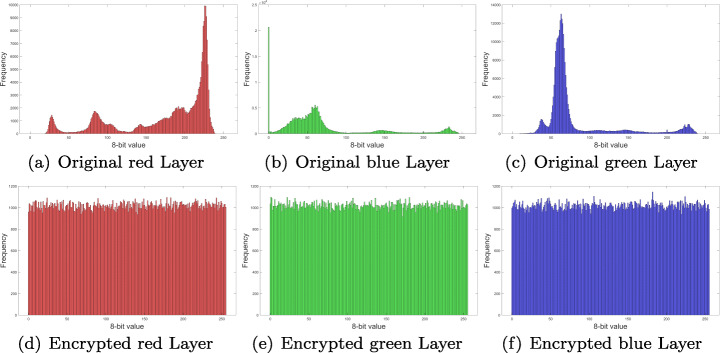
RGB layers histograms for Splash image from Fig. 7(c). Source: Own elaboration using MATLAB

**Fig. 15:**
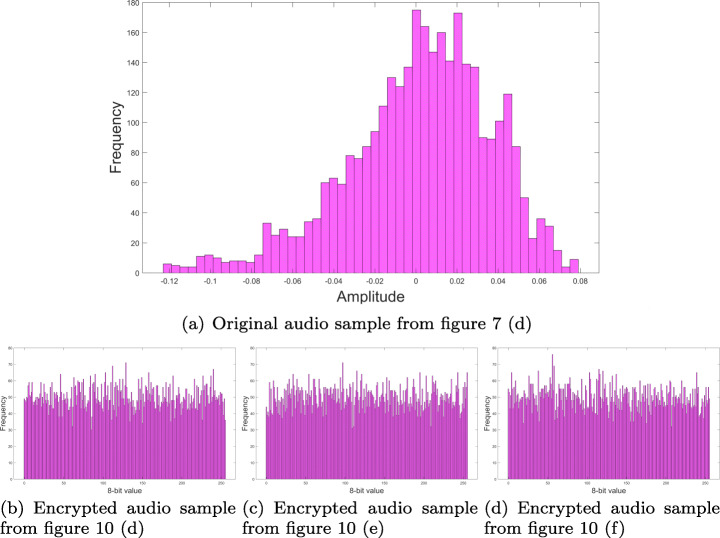
Audio samples histograms for audio sample from Fig. 10 o Source: Own elaboration using MATLAB

Distribution frequency histograms were plotted for each one of the RGB layers and audio channel in Fig. 7. Histograms in Figs. 12, 13, 14 and 15, show that encrypted information tends to be uniformly distributed, which is a good indicator for the encryption scheme. Also note, that audio sample histograms show different results based on the encrypted image.

On the other hand, *χ*^2^ test was used in order to establish the pixels distribution uniformity in encrypted image, a small value of this indicator means high pixel uniformity, which is desirable into a cryptographic process. The expression for this test is given by (20):

20 $$  \chi^{2}=\sum\limits_{i=0}^{255} \frac{(p_{i}-\bar{p})^{2}}{\bar{p}} $$

Where $\bar {p}$ represents the average frequency of all pixels $\frac {M\times N}{256}$ and *p*_*i*_ represents frequency of the *i* pixel in the image.

Table 3 summarizes *χ*^2^ values for original and encrypted images as well as some reference comparisons, the values obtained shows lower values for encrypted images and within the reference’s range.

**Table 3:** *χ*^2^ analysis for original and encrypted images. Source: Own elaboration

	Original			Encrypted		
	R	G	B	R	G	B
Peppers	491428	318382	213187	279.9721	286.8191	256.0783
Lena	254333	113929	344338	223.4926	279.8192	268.7801
Splash	605662	770974	1479241	269.4389	283.9328	241.4629
Jelly Beans	537503	349247	129762	263.7489	260.9411	236.5179
Mandrill	82839	142808	79942	248.1392	247.2922	284.1289
Tree	81371	57009	129824	226.0083	241.1531	280.1251
Gray	2933613	2933613	2933613	239.8315	229.2827	247.2589
Reference [31]	35300	32679	64067	258.8352	247.7227	208.7380
Reference [43]	160001	−	−	230.5898	−	−
Reference [42]	158875	−	−	247.4040	−	−
Reference [50]	−	−	−	249.9074	249.9074	249.9074
Reference [51]	−	−	−	262.9128	262.9128	262.9128

### Correlation analysis

Correlation analysis is a statistical measure that evaluates diffusion and confusion for an encryption algorithm [54]. Such analysis is based on (21).

21 $$  r_{xy}=\frac{cov(x,y)}{\sqrt{D(x)D(y)}} $$

Where *cov*(*x*,*y*), *D*(*x*), *D*(*y*) are given by (22), (23) and (24), respectively.

22 $$  cov(x,y)=\frac{1}{n}\sum\limits_{i=1}^{n}(x_{i}-E(x))(y_{i}-E(y)) $$

23 $$  D(x)=\frac{1}{n}\sum\limits_{i=1}^{n}(x_{i}-E(x))^{2} $$

24 $$  D(y)=\frac{1}{n}\sum\limits_{i=1}^{n}(y_{i}-E(y))^{2} $$

In equations above, *x*_*i*_ corresponds to *i*-th sample’s value, *y*_*i*_ is the adjacent value and *E*(*x*) is the mean given by (25).

25 $$  E(x)=\frac{1}{n}\sum\limits_{i=1}^{n}x_{i} $$

Correlation coefficients for encrypted images were obtained from around 10% random samples of total’s data in horizontal, vertical and diagonal directions. Each sample is obtained from the corresponding pixel associated to each layer, Table 4 shows results for encrypted images and some references taken into consideration.

**Table 4:** Correlation coefficients for images in Fig. 7(a)–(c)

		Original			Encrypted		
	Direction	R	G	B	R	G	B
	Vertical	0.9637	0.9814	0.9645	− 0.0057	− 0.0145	0.0054
Peppers	Horizontal	0.9682	0.9821	0.9683	− 0.0034	0.0011	− 0.0012
	Diagonal	0.9570	0.9682	0.9469	− 0.0031	0.0187	− 0.0055
	Vertical	0.9801	0.9706	0.9361	− 0.0063	0.0065	0.0111
Lena	Horizontal	0.9893	0.9826	0.9564	− 0.0054	− 0.0079	0.0035
	Diagonal	0.9689	0.9537	0.9145	− 0.0053	− 0.0020	0.0068
	Vertical	0.9935	0.9804	0.9817	0.0043	− 0.0042	− 0.0024
Splash	Horizontal	0.9946	0.9864	0.9772	0.0121	0.0040	− 0.0011
	Diagonal	0.9892	0.9714	0.9649	− 0.0020	− 0.0116	− 0.0024
	Vertical	0.9736	0.9722	0.9795	0.0009	− 0.0035	− 0.0030
Jelly Beans	Horizontal	0.9718	0.9728	0.9767	− 0.0024	0.0320	− 0.0004
	Diagonal	0.9532	0.9524	0.9627	0.0029	0.0097	0.0134
	Vertical	0.9230	0.8624	0.9038	0.0115	− 0.0072	− 0.0202
Mandrill	Horizontal	0.8632	0.7595	0.8773	− 0.0071	0.0069	0.0311
	Diagonal	0.8533	0.7331	0.8372	0.0004	0.0102	− 0.0007
	Vertical	0.9585	0.9685	0.9622	0.0044	− 0.0050	0.0250
Tree	Horizontal	0.9398	0.9469	0.9427	0.0124	0.0079	0.0041
	Diagonal	0.9117	0.9330	0.9248	0.0003	0.0218	− 0.0059
	Vertical	0.9962	0.9962	0.9962	0.0010	0.0051	0.0002
Gray	Horizontal	0.9998	0.9998	0.9998	− 0.0061	− 0.0095	− 0.0022
	Diagonal	0.9998	0.9998	0.9998	− 0.0119	0.0044	0.0003

Source: Own elaboration

Note that results present at Table 4 resemble those reported in other works with similar approaches as shown in Table 5.

**Table 5:** Correlation coefficients from similar approaches

	Encrypted Red channel		
	Vertical	Horizontal	Diagonal
Peppers	− 0.0057	− 0.0034	− 0.0031
Lena	− 0.0063	− 0.0054	− 0.0053
Splash	0.0043	0.0121	− 0.0020
Jelly Beans	0.0009	− 0.0024	0.0029
Mandrill	0.0115	− 0.0071	0.0004
Tree	0.0044	0.0124	0.0003
Gray	0.0010	− 0.0061	− 0.0119
Reference [47]	0.0003	− 0.0084	− 0.0089
Reference [31]	0.0021	0.0018	− 0.0195
Reference [33]	− 0.0024	0.0035	0.0014
Reference [26]	0.0009	− 0.0018	− 0.0039
Reference [29]	0.0033	0.0058	0.0010
Reference [32]	− 0.0012	0.0026	0.0013
Reference [56]	− 0.0021	0.0034	0.0012
Reference [43]	0.0020	− 0.0060	− 0.0004
Reference [42]	− 0.0059	− 0.0010	0.0072
Reference [50]	− 0.0076	− 0.0049	0.0093
Reference [51]	− 0.0040	− 0.0028	0.0057

For audio sample in Fig. 7(d), correlation analysis was done by comparing the left and right channels of original and encrypted samples. Correlation was also computed by comparing the original and encrypted version of each channel independently. The original audio correlation on both channels was found to be 0.2342 whereas encrypted samples test results are shown in Table 6, ‘L’ and ‘R’ represent the left and right channels respectively, ‘o’ refers to the original audio and ‘c’ denotes the ciphered audio.

**Table 6:** Correlation coefficients for audio sample of Fig. 7(c)

	Encrypted Audio Lc/Rc	Left channel Lo/Lc	Right channel Ro/Rc
Pepper	− 0.0017	0.0064	0.0343
Lena	− 0.0034	0.0049	0.0030
Splash	0.0041	0.0094	0.0047
Jelly Beans	− 0.0180	0.0007	− 0.0035
Mandrill	0.0124	− 0.0265	− 0.0002
Tree	0.0102	0.0038	0.0102
Gray	0,0079	0.0091	0.0168

Source: Own elaboration

According to Tables 4 and 6 results, it can be concluded that original images and audio sample presented a strong correlation (close to 1), whereas the correlation coefficients for the encrypted information were close to 0, values that are very similar to those presented at Table 5.

### Differential analysis

Differential analysis in multimedia files is measured by introducing small changes into the original information. For images case, one pixel value is modified randomly, whereas for an audio file a random sample is chosen and the least significant bit is changed by inverting its binary value, resulting into a new audio signal [22]. Once a small change is introduced, both the original and altered multimedia information are encrypted using the same key for comparison. Given that purpose, metrics such as Number of Pixels Change Rate (NPCR), Unified Average Changing Intensity (UACI) and Number of Sample Change Rate (NSCR) were computed for each RGB layer and audio channel. Expressions for these metrics are defined from (26) to (31).

26 $$  NPCR=\frac{{\sum}_{i,j}D(i,j)}{M\times N}\times 100\% $$

27 $$  D(i,j)= \begin{cases} 0, & C_{1}(i,j)=C_{2}(i,j) \\ 1, & \text{other case} \end{cases} $$

28 $$  UACI=\frac{1}{M\times N}\sum\limits_{i,j}\frac{| C_{1}(i,j)-C_{2}(i,j)|}{255}\times 100\% $$

Where *C*_1_(*i*,*j*), *C*_2_(*i*,*j*) are two encrypted images, the first one corresponds to original image and the second one to the same image with one altered pixel; *M* and *N* correspond to image’s size.

29 $$  NSCR=\frac{{\sum}_{i}D(i)}{L}\times 100\% $$

30 $$  D(i)= \begin{cases} 0, & A_{i}\ne A_{i}^{\prime} \\ 1, & \text{other case} \end{cases} $$

31 $$  UACI^{\prime}=\frac{1}{L}\sum\limits_{i}\frac{| A_{i}\ne A_{i}^{\prime}|}{65535}\times 100\% $$

Where *A* and $A^{\prime }$ are two encrypted audio signals, first one being the original sample and the other one the audio with one altered bit; *L* corresponds to audio vector length.

Results obtained are presented in Tables 7 and 8, indicating that image layers and audio samples meet the optimal expected values for the NPCR, UACI and NSCR, UACI’, respectively, which are 99.61*%*, 33.46% and 100*%*, 33.3% [22, 54].

**Table 7:** Differential analysis for proposed implementation images and references

	NPCR			UACI		
	R	G	B	R	G	B
Peppers	0.9962	0.9960	0.9960	0.3347	0.3346	0.3351
Lena	0.9962	0.9960	0.9960	0.3338	0.3345	0.3355
Splash	0.9962	0.9960	0.9960	0.3351	0.3350	0.3351
Jelly Beans	0.9957	0.9962	0.9957	0.3362	0.3347	0.3361
Mandrill	0.9961	0.9960	0.9962	0.3359	0.3347	0.3355
Tree	0.9961	0.9960	0.9966	0.3364	0.3371	0.3360
Gray	0.9959	0.9959	0.9961	0.3350	0.3360	0.3356
Reference [47]	0.9958	0.9962	0.9961	0.3370	0.3335	0.3345
Reference [31]	0.9943	0.9936	0.9942	0.2838	0.3928	0.3289
Reference [33]	0.9961	0.9963	0.9964	0.3357	0.3334	0.3340
Reference [26]	0.9960	0.9959	0.9964	0.3306	0.3056	0.2760
Reference [29]	0.9962	0.9962	0.9967	0.3347	0.3343	0.3346
Reference [32]	0.9957	0.9951	0.9959	0.3332	0.3341	0.3344
Reference [50]	0.9961	0.9961	0.9961	0.3379	0.3379	0.3379
Reference [56]	0.9470	−	−	0.3328	−	−
Reference [43]	0.9960	−	−	0.3345	−	−

Source: Own elaboration

**Table 8:** Differential analysis for proposed implementation audio samples

	NSCR		UACI’	
	Left channel	Right channel	Left channel	Right channel
Peppers	0.9961	0.9958	0.3328	0.3297
Lena	0.9969	0.9964	0.3369	0.3346
Splash	0.9948	0.9944	0.3346	0.3319
Jelly Beans	0.9965	0.9962	0.3332	0.3380
Mandrill	0.9954	0.9964	0.3362	0.3314
Tree	0.9956	0.9957	0.3376	0.3340
Gray	0.9957	0.9961	0.3336	0.3310

Source: Own elaboration

Values obtained in this paper are similar to those of other references presented in Table 7. These results shows that with a minimum variation in original information, different encrypted information is obtained, translating into a high resistance to differential attacks.

### Encryption quality analysis

This type of analysis measures the difference between repetition frequency for each pixel value of plaintext and encrypted image, expression given in (32) describes encryption measure quality (*EQ*), where *o*_*i*_(*P*) and *o*_*i*_(*C*) correspond to the number of pixel with *i* intensity in original and encrypted images respectively.

32 $$  EQ=\frac{1}{256}\sum\limits_{i=1}^{255}|o_{i}(P)-o_{i}(C)| $$

According to reference [1] the maximum value for EQ is given by expression (33), being *N*,*M* the image’s width and height.

33 $$  EQ_{max}=\frac{510\times N\times M}{256^{2}} $$

Table 9 shows encryption quality analysis results obtained from the proposed algorithm, which complies with expression (33) and even shows superior measures than those reported in [43].

**Table 9:** Encryption quality analysis results for proposed model and references

	R	G	B
Pepper	832	612	946
Lena	807	583	1016
Splash	930	1039	1277
Jelly Beans	330	308	260
Mandrill	510	705	478
Tree	186	182	245
Gray	1877	1878	1876
Reference[43]	179	−	−

Source: Own elaboration

### Key sensitivity

**Fig. 16:**
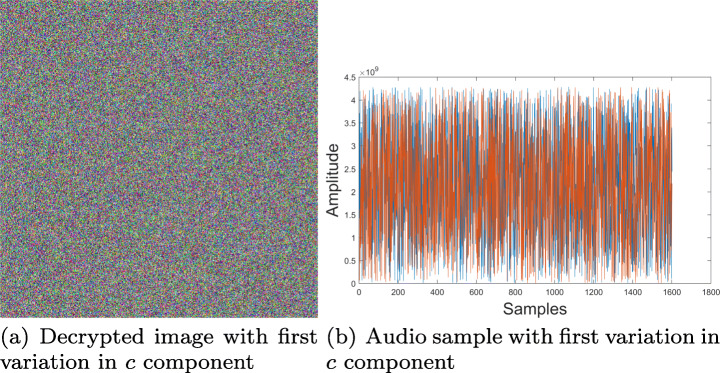
Decrypted sample with variation *c* = − 0.310 − 0.041*i* − 0.325*j* + 0.56000000001*k*. Source: Own elaboration using MATLAB

**Fig. 17:**
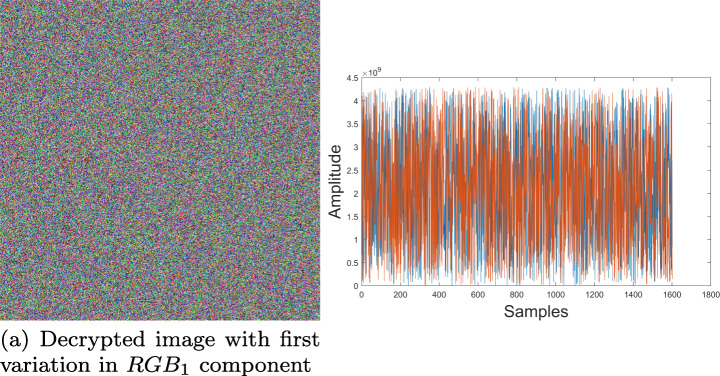
Decrypted sample with variation *RBG*_1_ = (0.210001,0.6,0.4). Source: Own elaboration using MATLAB

In order to evaluate the key sensitivity in proposed cryptosystem, elements used in Fig. 7(b) and (d) were encrypted with Fig. 8 key. Subsequently, small changes were introduced to arbitrary key parameters such as the *RGB*_1_ vector and constant *c* of *f*(*z*) function. In both scenarios using such non-significant variations, decryption of original information was unsuccessful. Results obtained are shown in Figs. 16 and 17, indicating that proposed cryptosystem is sensitive to small key changes with an approximate precision of 10^− 11^ ≈ 2^− 33^, result being useful for key space calculation in next section.

As it can be seen on results obtained, knowing even one of the parameters isn’t enough to decipher the original multimedia file, therefore it is possible to assure that the proposed system is resistant to brute-force attacks. In addition to that, a small variation in any of key parameters, causes totally different results. This was experimentally corroborated by calculating the correlation between two key images with insignificant changes in their parameters, obtaining an average correlation of 0.0153, which implies that even if the algorithm is known, but there is no certainty about all parameters used in the encryption process, it is impossible to recover the original information.

### Key space analysis

According to the proposal, system’s key space can be deduced from CSPRNG seed, *snk* shift and the different parameters involved in key generation such as c, pc (128 bit quaternions given by Section 5.6); *RGB*_1_, *RGB*_2_, *RGB*_3_, *RGB*_4_ 8 bits vectors for each input in $\mathbb {R}^{3}$; and *n*, *Fr* integers in the order of 5 and 3 bits respectively, indicating that proposed algorithm key space is larger than 2^360^ = 2^2∗128 + 12∗8 + 5+ 3^.

### Entropy analysis

An entropy value means the level of resistance to statistical attacks as well as the randomness level of encrypted information, this value is calculated using (34), where *M* is the maximum information value to be analyzed, *m*_*i*_ is the *i*-th information’s value and *p*(*m*_*i*_) the *m*_*i*_ probability of occurrence [3]. In the case of RGB images, with 256 possible values for each layer, encrypted image entropy must be close to 8, which also applies for audio samples, this kind of analysis indicates a high resistance to this kind of attack [22, 54].

34 $$  H(m)=-\sum\limits_{i=1}^{M}p(m_{i})\log_{2}(p(m_{i})) $$

Values obtained in this proposal for image layers and audio channels are close to 8, showing that ciphered information has a high confusion level and are very similar to results found on different references, indicating a high disorder degree. Results are presented in Table 10.

**Table 10:** Entropy values for proposed models and references. Source: Own elaboration

	R	G	B	Right channel	Left channel
Peppers	7.9973	7.9973	7.9968	7.9717	7.9687
Lena	7.9973	7.9970	7.9970	7.9717	7.9687
Splash	7.9972	7.9972	7.9972	7.9717	7.9687
Jelly Beans	7.9950	7.9959	7.9951	7.9662	7.9667
Mandrill	7.9969	7.9976	7.9970	7.9727	7.9650
Tree	7.9952	7.9946	7.9948	7.9694	7.9668
Gray	7.9974	7.9968	7.9971	7.9654	7.9634
Reference [47]	7.9974	7.9969	7.9968	−	−
Reference [31]	7.9875	7.9880	7.9876	−	−
Reference [33]	7.9972	7.9972	7.9972	−	−
Reference [26]	7.9992	7.9993	7.9994	−	−
Reference [29]	7.9973	7.9975	7.9975	−	−
Reference [32]	7.9973	7.9975	7.9975	−	−
Reference [50]	7.9994	7.9994	7.9994	−	−
Reference [51]	7.9986	7.9986	7.9986	−	−
Reference [56]	7.9987	−	−	−	−
Reference [43]	7.9993	−	−	−	−
Reference [42]	7.9993	−	−	−	−

### Local entropy analysis

Sometimes Shannon entropy is not a reliable indicator of randomness level, for this reason it is necessary to calculate different local entropies that reflect a more accurate randomness level of pixels [45].

Local Shannon entropy takes a *N* finite blocks number *m*_*l*_, *l* = 1,...,*N* choosen randomly to calculate the corresponding entropy value, then the average Shannon entropies are calculated and synthesized in expression 35.

35 $$  \overline{H_{L}}(m)=\sum\limits_{l=1}^{N}\frac{H(m_{l})}{N} $$

*H*(*m*_*l*_) is given by (34).

Table 11 presents local entropy values obtained for RGB layers in each image showing that results are close to 8, and also within the range from other references.

**Table 11:** Local entropy values for proposed model

	R	G	B
Peppers	7.9804	7.9808	7.9798
Lena	7.9811	7.9806	7.9808
Splash	7.9802	7.9797	7.9797
Jelly Beans	7.9281	7.9292	7.9264
Mandrill	7.9798	7.9812	7.9808
Tree	7.9297	7.9280	7.9263
Gray	7.9810	7.9797	7.9805
Reference [51]	7.9063	7.9063	7.9063
Reference [43]	7.9030	−	−
Reference [42]	7.9020	−	−

Source: Own elaboration

### Robustness analysis

**Fig. 18:**
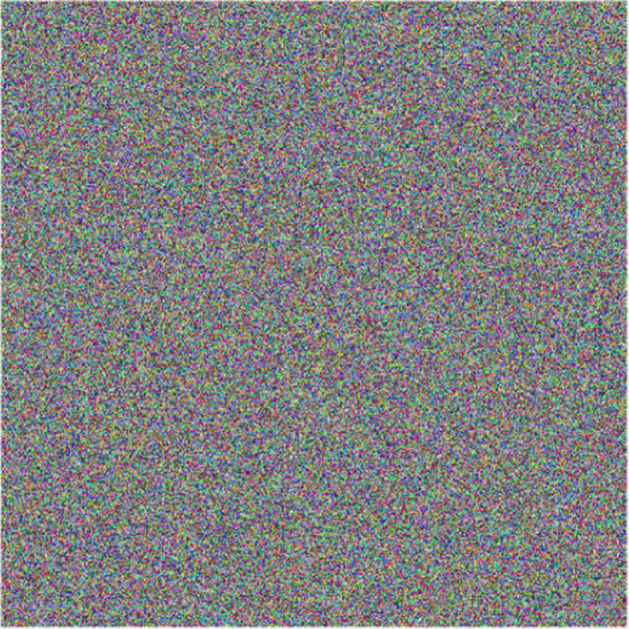
Decryption image with 1-bit noise. Source: Own elaboration using MATLAB

Salt & pepper and occlusion tests were performed on proposed algorithm demonstrating a high sensitivity towards any disturbance on encrypted information, due to the fact that by altering a single bit and having all the necessary parameters to start the decryption process it was not possible to recover the original file as shown in Fig. 18. For example the robustness for an image of 512 × 512 size, when a single bit change is done corresponds to $\frac {1}{32\times 512\times 512}=1.192 \times 10^{-7}$ bits where factor 32 is associated to the three RGB layers and audio channels altogether using 8 bits precision each, such fact is perceived as a strength for the proposed encryption method, however, this entails that a mechanism must be implemented in order to fix errors inherent to the communication channel, guarantying full reliability and ensure that receiver can access the multimedia file sent, an analysis with similar results is presented in [56].

Peak signal-to-noise ratio (PSNR) is a measure that indicates the noise that can be added in a decryption process, a larger value in this indicator evidences a lower noise proportion in the recovered information. Equation 36 defines PSNR, in the case of this proposal, the mean square error (*MSE*) is zero, because the two images are identical, therefore PSNR has a tendency to infinity, indicating that during the decryption process there are no alterations in original information.

36 $$  PSNR=10\log{\frac{255^{2}}{MSE}} $$

**Fig. 19:**
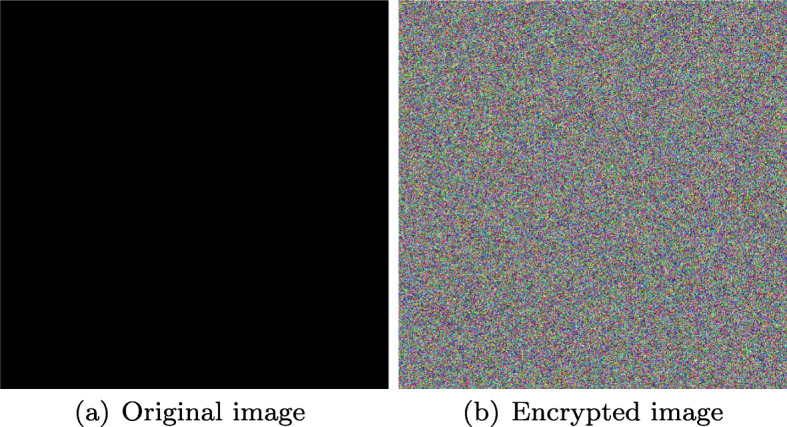
Encrypted black image. Source: Own elaboration using MATLAB

**Fig. 20:**
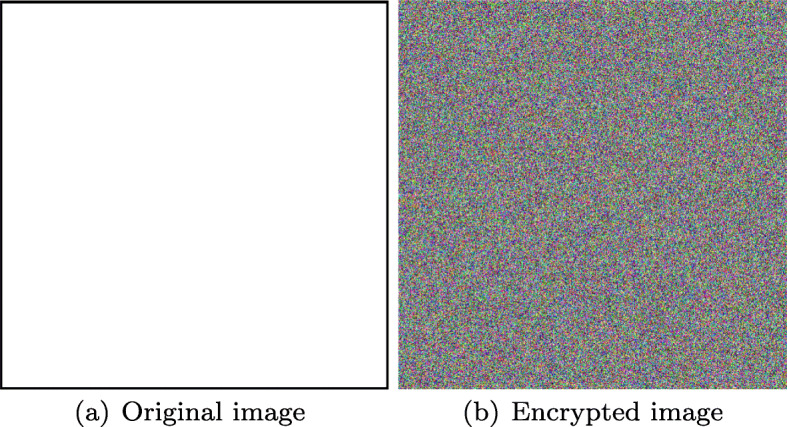
Encrypted white image. Source: Own elaboration using MATLAB

Also Figs. 19 and 20 show that for black or white images along with audio sample from Fig. 7(d), encryption process can be performed successfully.

### Resistance attack analysis

Four common cipher attack types are Ciphertext-Only, known-plaintext, chosen-ciphertext and chosen-plaintext, with chosen-plaintext being the most effective one. According to [14], a system resistant to this type of attack is resistant to the others, regardless of plaintext. Information is masked using a CSPRNG to avoid frequency attacks, followed by the snake, which has high sensitivity since a small change in the masked image causes alterations in all layers, these two operations are linear, while the matrix and quaternion product are not. In case of a possible plaintext attack it is necessary to carry out the inversible matrix search, which implies a high amount of computational resources.

Furthermore, proposed algorithm also features brute force attack resistance because key is an image generated from 360 bits according to Section 5.7 and include other security layers such as the snake shift *NM*, residual matrix product $2^{8N^2}2^{8M^2}$ and the CSPRNG seed at least of 2^128^ bits.

### Computational complexity

**Fig. 21:**
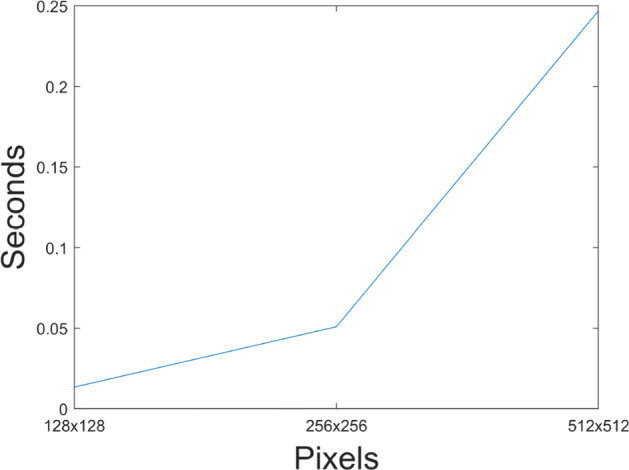
Computational complexity for lena. Source: Own elaboration using MATLAB

Computational complexity of an algorithm refers to the time measurement an algorithm execution takes with respect to data size, which is an indicator of the algorithm’s efficiency and represents its computational boundary. In this article using the computer described in Section 4, and Lena image with different resolutions (128 × 128,256 × 256,512 × 512), the time taken by algorithm respectively is presented in Fig. 21 and Table 12, highlighting that this proposal has a quadratic complexity *O*(*n*^2^), nevertheless as information increases, time increases proportionally to matrix size.

**Table 12:** Time complexity for different Lena sizes

Size	Time (s)	Time(s) / Total bits
128	0,0134026	9, 42 ⋅ 10^− 07^
256	0,0509530	7, 77 ⋅ 10^− 07^
512	0,2470514	8, 18 ⋅ 10^− 07^

Source: Own elaboration

## Conclusions

An encryption method applied to multimedia files was proposed based on the properties of chaotic attractors in a 3D space, using an image generated from the Julia Quatertion attractor as a key ensuring with this a wide space and sensibility.

Quaternion numbers were used to take cryptographic advantage of various properties such as the non-commutative product, which avoids typical issues with traditional encryption schemes such as the discrete logarithm problem and faster calculation for rotation operations. Also using this set of numbers made it possible to design an integrated structure for audio and images, ensuring that a variation in any part of the process produces changes in the whole information.

Using the congruence theory properties and modulus 257 residual matrices, it was achieved that small changes in pixels generate totally different keys increasing its sensibility. In addition, the use of residual matrices made it possible to take advantage of their inverse and non-commutational product, making it easier to include the modified information within the $\mathbb {Z}_{257}$ ring.

Combination of quaternions and residual matrices in this proposal contributed, firstly, by adding a highly sensitive, fast and nonlinear layer, which led to find safety and performance indicators comparable with similar works, this allows application of the proposed method in real world environments. On the other hand, computation time was reduced by the usage of dynamic programming and existence of an unique inverse element in $\mathbb {Z}_{257}$ ring.

Aiming at improving results of this proposal, future work should explore the use of other security layers, chaotic images and attractors along with its behavior on 3D environments in order to improve the algorithm and its security, especially as it related to salt & pepper and occlusion tests. In the same way the proposed algorithm could be implemented using parallel programming reducing its computational requirements.

## Data Availability

The datasets analysed during the current study are available in the USC-SIPI Image repository following the link https://sipi.usc.edu/database/. For key generation PovRay was used, a Free software with a native Julia Quaterion Fractal implementation, for more information go to https://www.povray.org/documentation/view/3.7.0/280/. The authors declare that data supporting findings of this study are available within the article. Also, data is available from authors upon reasonable request.

## References

[1] Abu Taha M, Hamidouche W, Sidaty N, Viitanen M, Vanne J, El Assad S, Deforges O (2020) Privacy protection in real time hevc standard using chaotic system. Cryptography 4(2). 10.3390/cryptography4020018. https://www.mdpi.com/2410-387X/4/2/18

[2] Ahmad I, Shin S (2021). A novel hybrid image encryption–compression scheme by combining chaos theory and number theory. Signal Process Image Commun.

[3] Albahrani E (2017) A new audio encryption algorithm based on chaotic block cipher. pp 22–27. 10.1109/NTICT.2017.7976129

[4] Anand R, Bajpai G, Bhaskar V (2009) Real-time symmetric cryptography using quaternion julia set. International Journal of Computer Science and Network Security

[5] Anghelescu P (2012) Encryption of multimedia medical content using programmable cellular automata. In: World congress on internet security (WorldCIS-2012), pp 11–16

[6] Banik A, Shamsi Z, Laiphrakpam DS (2019). An encryption scheme for securing multiple medical images. Journal of Information Security and Applications.

[7] Bassham LE III, Rukhin AL, Soto J, Nechvatal JR, Smid ME, Barker EB, Leigh SD, Levenson M, Vangel M, Banks DL et al (2010) Sp 800-22 rev. 1a. a statistical test suite for random and pseudorandom number generators for cryptographic applications. National Institute of Standards & Technology

[8] Boussif M, Aloui N, Cherif A (2019) Images encryption algorithm based on the quaternion multiplication and the xor operation. Multimed Tools Appl 78. 10.1007/s11042-019-08108-9

[9] Chen B, Yu M, Tian Y, Li L, Wang D, Sun X (2018). Multiple-parameter fractional quaternion fourier transform and its application in colour image encryption. IET Image Process.

[10] Dahua X, Kuo C C J (2007) Multimedia encryption with joint randomized entropy coding and rotation in partitioned bitstream. EURASIP Journal on Information Security 2007. 10.1155/2007/35262

[11] Devaney R (1948) An introduction to chaotic dynamical systems. Wesley Publishing Company

[12] Dzwonkowski M, Rykaczewski R (2018) Secure quaternion feistel cipher (s-qfc) for dicom images. IEEE Transactions on Image Processing PP 1–1. 10.1109/TIP.2018.2868388 10.1109/TIP.2018.2868388 30183633

[13] Dzwonkowski M, Papaj M, Rykaczewski R (2015). A new quaternion-based encryption method for dicom images. IEEE Trans Image Process.

[14] Fang P, Liu H, Wu C (2021). A novel chaotic block image encryption algorithm based on deep convolutional generative adversarial networks. IEEE Access.

[15] Kamlofsky J, Hecht JP, Izzi O, Masuh S A diffie-hellman compact model over non-commutative rings using quaternions. 10.13140/RG.2.1.4063.1760

[16] Kasianchuk M, Yakymenko I, Pazdriy I, Melnyk A, Ivasiev S (2017) Rabin’s modified method of encryption using various forms of system of residual classes. In: 2017 14th international conference the experience of designing and application of cad systems in microelectronics (CADSM), pp 222–224. 10.1109/CADSM.2017.7916120

[17] Khalil M (2017) Integrating and securing video, audio and text using quaternion fourier transform. International Journal of Communication Networks and Information Security 9

[18] Khan M, Masood F, Alghafis A (2020) Secure image encryption scheme based on fractals key with fibonacci series and discrete dynamical system. Neural Comput & Applic 32. 10.1007/s00521-019-04667-y

[19] Kuipers J B (2000). In: Quaternions and rotation sequences. In: Proceedings of the International Conference on Geometry, Integrability and Quantization. Coral Press Scientific Publishing, Sofia, pp 127–143, 10.7546/giq-1-2000-127-143

[20] Kumar G, Saini H (2017). Novel noncommutative cryptography scheme using extra special group. Security and Communication Networks.

[21] Li Q, Wang X, Ma B, Wang X, Wang C, Gao S, Shi Y (2021) Concealed attack for robust watermarking based on generative model and perceptual loss. IEEE Trans Circuits Syst Video Technol 1–1. 10.1109/TCSVT.2021.3138795

[22] Lima J, Da Silva Neto E (2015) Audio encryption based on the cosine number transform. Multimed Tools Appl 75. 10.1007/s11042-015-2755-6

[23] Liu H, Wang X (2010). Color image encryption based on one-time keys and robust chaotic maps. Computers & Mathematics with Applications.

[24] Liu H, Wang X (2011). Color image encryption using spatial bit-level permutation and high-dimension chaotic system. Opt Commun.

[25] Liu H, Wang X, kadir A (2012). Image encryption using dna complementary rule and chaotic maps. Appl Soft Comput.

[26] Liu Z, Wu C, Wang J, Hu Y (2019). A color image encryption using dynamic dna and 4-d memristive hyper-chaos. IEEE Access.

[27] Masood F, Ahmad J, Shah S A, Jamal S S, Hussain I (2020) A novel hybrid secure image encryption based on julia set of fractals and 3d lorenz chaotic map. Entropy 22. 10.3390/e22030274 10.3390/e22030274 PMC7516729 33286048

[28] Min L, Zhang L, Zhang Y (2013) A novel chaotic system and design of pseudorandom number generator. In: 2013 fourth international conference on intelligent control and information processing (ICICIP), pp 545–550. 10.1109/ICICIP.2013.6568135

[29] Mohamed, ElKamchouchi, Moussa (2020). A novel color image encryption algorithm based on hyperchaotic maps and mitochondrial dna sequences. Entropy.

[30] Nagase T, Komata M, Araki T (2004) Secure signals transmission based on quaternion encryption scheme. In: 18th international conference on advanced information networking and applications, 2004. AINA 2004, vol 2, pp 35–38. 10.1109/AINA.2004.1283751

[31] Niu Y, Sun X, Zhang C, Liu H (2020) Anticontrol of a fractional-order chaotic system and its application in color image encryption. Math Probl Eng 2020. 10.1155/2020/6795964

[32] Niyat A, Moattar M (2020) Color image encryption based on hybrid chaotic system and dna sequences. Multimed Tools Appl 79. 10.1007/s11042-019-08247-z

[33] Pak C, An K, Jang P, Kim J, Kim S (2019) A novel bit-level color image encryption using improved 1d chaotic map. Multimed Tools Appl 78. 10.1007/s11042-018-6739-1

[34] Risqi YSS, Windarta S (2017) Statistical test on lightweight block cipher-based prng. 10.1109/TSSA.2017.8272925

[35] Rodriguez IF, Amaya EI, Suarez CA, Moreno JD (2017). Algoritmo de Encriptacion de Imagenes Utilizando el Atractor Caotico de Lorenz. Ingeniería.

[36] Salem M, Abboud A, Yildirim R (2022). A visual cryptography-based watermarking approach for the detection and localization of image forgery. Electronics.

[37] Sokouti M et al (2016) Medical image encryption: An application for improved padding based ggh encryption algorithm. Open Med Inform J. 10.2174/1874431101610010011 10.2174/1874431101610010011 PMC5090780 27857824

[38] Soni R, Johar A, Soni V (2013) An encryption and decryption algorithm for image based on dna. In: 2013 international conference on communication systems and network technologies, pp 478–481. 10.1109/CSNT.2013.105

[39] Su Z, Zhang G, Jiang J (2012) Multimedia security: A survey of chaos-based encryption technology. In: Karydis I (ed) Multimedia, IntechOpen, Rijeka, chap 5, DOI 10.5772/36036

[40] Tawalbeh L, Mowafi M, Aljoby W (2013). Use of elliptic curve cryptography for multimedia encryption. IET Inf Secur.

[41] Valluri M, Narayan S (2016) Quaternion public key cryptosystems. 10.1109/WCICSS.2016.7882612

[42] Wang X, Liu P (2022). A new full chaos coupled mapping lattice and its application in privacy image encryption. IEEE Trans Circuits Syst I Regul Pap.

[43] Wang X, Zhang M (2021). An image encryption algorithm based on new chaos and diffusion values of a truth table. Inf Sci.

[44] Wang X, Yang L, Liu R, Kadir A (2010). A chaotic image encryption algorithm based on perceptron model. Nonlinear Dyn.

[45] Wu Y, Zhou Y, Saveriades G, Agaian S, Noonan JP, Natarajan P (2013). Local shannon entropy measure with statistical tests for image randomness. Inf Sci.

[46] Xian Y, Wang X, Teng L (2021) Double parameters fractal sorting matrix and its application in image encryption. IEEE Trans Circuits Syst Video Technol 1–1. 10.1109/TCSVT.2021.3108767

[47] Xiang H, Liu L (2020). An improved digital logistic map and its application in image encryption. Multimed Tools Appl.

[48] Xing X, Zhu Y, Mo Z, Sun Z, Liu Z (2015). A novel perceptual hashing for color images using a full quaternion representation. KSII Trans Internet Inf Syst.

[49] Ye HS, Zhou N, Gong L (2020). Multi-image compression-encryption scheme based on quaternion discrete fractional hartley transform and improved pixel adaptive diffusion. Signal Process.

[50] Yousif SF, Abboud AJ, Radhi HY (2020). Robust image encryption with scanning technology, the el-gamal algorithm and chaos theory. IEEE Access.

[51] Yousif SF, Abboud AJ, Alhumaima RS (2022). A new image encryption based on bit replacing, chaos and dna coding techniques. Multimedia Tools Appl.

[52] Yu C, Li J, Li X, Ren X, Gupta B B (2018) Four-image encryption scheme based on quaternion fresnel transform, chaos and computer generated hologram. Multimed Tools Appl 77. 10.1007/s11042-017-4637-6

[53] Zhang X, Hu Y (2021). Multiple-image encryption algorithm based on the 3d scrambling model and dynamic dna coding. Optics & Laser Technology.

[54] Zhang X, Seo S, Wang C (2018). A lightweight encryption method for privacy protection in surveillance videos. IEEE Access.

[55] Zheng J, Hu H (2022). A highly secure stream cipher based on analog-digital hybrid chaotic system. Inf Sci.

[56] Zhu S, Zhu C (2019). Plaintext-related image encryption algorithm based on block structure and five-dimensional chaotic map. IEEE Access.

